# Predicting the Immune Microenvironment and Prognosis with a NETosis-Related lncRNA Signature in Head and Neck Squamous Cell Carcinoma

**DOI:** 10.1155/2022/3191474

**Published:** 2022-09-12

**Authors:** Xiaohua He, Yinglu Xiao, Shan Liu, Ruyan Deng, Zhiming Li, Xianying Zhu

**Affiliations:** ^1^State Key Laboratory of Oncology in South China, Collaborative Innovation Center for Cancer Medicine, Sun Yat-sen University Cancer Center, Guangzhou, China; ^2^Department of Medical Oncology, Sun Yat-sen University Cancer Center, Sun Yat-sen University, Guangzhou, China; ^3^Department of Intensive Care Unit, Sun Yat-sen University Cancer Center, Sun Yat-sen University, Guangzhou, China

## Abstract

**Background:**

The mechanistic aspects of the involvement of long noncoding RNAs (lncRNAs) in NETosis, the process of neutrophil extracellular trap (NET) formation in head and neck squamous cell carcinoma (HNSCC), lack comprehensive elucidation. The involvement of these molecules in the immune microenvironment and plausible HNSCC prognosis remain to see the light of the day. The plausible functioning of NETosis-related lncRNAs with their plausible prognostic impact in HNSCC was probed in this work.

**Methods:**

The scrutiny of lncRNAs linked to NETosis entailed the probing of twenty-four genes associated with the process employing Pearson's correlation analysis on HNSCC patients' RNA sequencing data from The Cancer Genome Atlas (TCGA) database. The application of univariate, least absolute shrinkage and selection operator (LASSO), and multivariate Cox regression analyses yielded a NETosis-related lncRNA signature that was subjected to probing for its suitability in prognosis employing survival and nomogram analyses.

**Results:**

The NETosis-related lncRNA signature inclusive of five lncRNAs facilitated patients to be segregated as high-risk and low-risk groups with the former documenting a poor prognosis. Regression unearthed that the risk score was an independent factor for prognosis. The receiver operating characteristic (ROC) or receiver operating characteristic curve analysis documented a one-year area under time-dependent ROC curve (AUC) value of 0.711 that is corroborative of the accuracy of this signature. Additional probing documented an evident enriching of immune-linked pathways in the low-risk patients, while the high-risk patients documented an immunologically “cold” profile as per the infiltration of immune cells. We verified lncRNA expression from our NETosis-related lncRNA signature in vitro, which reflects the reliability of our model to a certain extent. Moreover, we also verified the function of the lncRNA. We found that LINC00426 contributes to the innate immune cGAS-STING signaling pathway, which explain to some extent the role of our prognostic model in predicting “hot” and “cold” tumors.

**Conclusions:**

The plausible prognostic relevance of the NETosis-related lncRNA signature (with five lncRNAs) emerges that is suggestive of its promise in targeting HNSCC.

## 1. Introduction

HNSCCs arise from the mucosal epithelium in the oral cavity, pharynx, and larynx and occupy the sixth position in global cancer incidence [1]. The most ubiquitously implicated risk factors for HNSCC encompass oncogenic human papillomavirus (HPV) infection, tobacco smoke, and excessive alcohol consumption [2]. HNSCC is remarkably heterogeneous for the anatomical location of cell origination, various etiologies, and carcinogenic mechanisms [3]. Most patients receive a late-stage HNSCC diagnosis without a clinical history of premalignancy [1]. Notwithstanding expanding surgical and nonsurgical approaches (inclusive of the radiotherapy, chemotherapy, and immunotherapy), the clinical prognosis of HNSCC still remains a roadblock, with the 5-year survival rate below 50% [4, 5]. Therefore, the probing of several plausible prognostic markers that accurately predict the outcome of HNSCC emerges as vital to assist the delineation of individualized treatment plans.

Neutrophil extracellular traps (NETs) are web-like DNA structures coated with histones, proteases, and granular and cytosolic proteins [6] and are released by neutrophils to trap microorganisms, and the process of their formation is referred to as NETosis [7]. The possible involvement of NETs in noninfectious diseases, such as autoimmunity, coagulation, acute injuries, and cancer, has been documented [6]. Its involvement in increased primary tumor growth, metastasis, and complications like venous thromboembolism in malignancies has also been probed [8]. It has been corroborated that NET extrusion induced by tumor-secreted CXCR1 and CXCR2 ligands exerts a protective effect on the malignancies from the cytotoxicity of natural killer (NK) cells and T cells [9]. The augmentation of the cell cycle to boost metastasis within the bloodstream by NETs to expand the metastatic potential of circulating tumor cells is also known [10]. Yang et al. demonstrated that the DNA component of NETs (NET-DNA) promotes cancer metastasis via the transmembrane protein CCDC25 [11]. However, studies probing the role of NETosis in HNSCC are few. Li et al. found that a hypercoagulable state is driven in oral squamous cell carcinoma via systemic inflammation to stimulate neutrophils to prime and release NETs [12]. While a recent study documented the scrutiny of a NET-related gene signature for predicting non-small-cell lung cancer prognosis [13], the role and functioning of NETosis warrants more research. Therefore, it is meaningful to discern novel NETosis-linked biomarkers to recognize the molecular mechanistic aspects of NETosis for prognosis prediction in HNSCC patients.

lncRNAs are RNAs exceeding 200 nucleotides in length and do not participate in protein coding but are involved in controlling gene expression [14]. In lung cancer, the involvement of lncRNAs to regulate NETs is known [13]. However, the probing of NETosis-associated lncRNAs in HNSCC is yet to see the light of the day making the prognostic value of NETosis-associated lncRNAs unclear.

Immunotherapy has revolutionized cancer treatment over the past two decades, mostly employing immune checkpoint blockade (ICB) approaches. As of 2019, ICB (pembrolizumab, an IgG4 humanized antibody to PD-1) was approved as first-line or subsequent therapy of recurrent or metastatic squamous cell carcinoma of the head and neck [15]. The tumor microenvironment is vitally linked to the response to ICB. ICB efficacy is poor in “cold” tumors documenting lower PD-L1 levels in tumor cells, macrophages, and immune cells [4]. The conversion of these “cold” tumors into “hot” ones for ICB therapy in HNSCC can augment the response [16]. Although the sensitization of tumors to immunotherapy (PD-1 + CTLA-4 dual checkpoint blockade) by NETosis inhibition has been documented recently [9], such studies are still limited. The probing of the relationship between NETosis and the tumor immune microenvironment to further comprehend “cold” HNSCC is warranted to facilitate optimal treatment systems for “cold” HNSCC.

This work was aimed at scrutinizing NETosis-related lncRNAs in HNSCC to comprehend the molecular and signaling pathways of this phenomenon in this malignancy and predict the prognosis in these patients. In addition, the links between NETosis and tumor immune microenvironment were further probed to provide a speculative basis in “cold” HNSCC therapy.

## 2. Materials and Methods

### 2.1. Patient Details

The RNA sequencing data and patient characteristics of HNSCC patients (502 malignant and 44 normal samples) were sourced from the TCGA database (https://portal.gdc.cancer.gov/repository). The clinicopathological attributes were inclusive of age, gender, smoking status, HPV status, tumor grade, tumor stage, survival time, and survival status. Following the exclusion of the normal samples (*n* = 44) and a patient with the overall survival (OS) missing, 499 patients documenting complete survival and sequencing data were enrolled in this work. Figure S1 is illustrative of the workflow employed.

### 2.2. Identifying NETosis-Related lncRNAs

Firstly, 24 NETosis-associated genes were identified by searching literature (Table S1) [9, 17–25]. lncRNA and protein-coding gene annotations in the Ensembl human genome browser GRCh38.p13 (http://asia.ensembl.org/index.html) then ensued [26]. The correlation between the lncRNAs and the expression of NETosis-associated genes was probed employing Pearson's correlation coefficients. NETosis-related lncRNAs were determined at *P* < 0.001 and |*R*| > 0.4.

### 2.3. Establishment and Validation of the NETosis-Related lncRNAs Prognostic Signature

This entailed the random assignment (2 : 1) of 499 patients into a training cohort and a validation cohort. NETosis-related lncRNAs for prognosis were first scored employing univariate Cox regression analysis of the patients' survival data in the training cohort (*P* < 0.05). LASSO Cox regression ensued of these prognostic NETosis-related lncRNAs to diminish the chance of overfitting as much as possible. Subsequent application of multivariate analyses facilitated the indication of the candidate lncRNAs significantly involved in OS prognosis prediction. Five relevant NETosis-related lncRNAs were identified for the prognostic model as per the lowest Akaike information criterion (AIC) value. The risk scores of the HNSCC patients were obtained by the normalized lncRNA expression levels and the corresponding regression coefficients. This entailed the following formula for its computation (risk score = *β*gene(1) × EXPgene(1) + *β*gene(2) × EXPgene(2) + ⋯+*β*gene(*n*) × EXPgene(*n*)) with the discerned lncRNA expression level as EXPgene and its multivariate Cox regression analysis coefficient as *β*. The median value of the risk score was employed to categorize patients in the training cohort into high-risk (≥ median number) and low-risk (<median number) groups. The following tests ensued to corroborate the signature: intergroup OS was scored by Kaplan-Meier analysis with the “survival” and “survminer” R package. The prediction accuracy was probed by the “survival ROC” R package employing time-dependent receiver operating characteristic (ROC) curve analysis. The scrutiny of the utility of this signature as an independent prognostic factor as opposed to other clinical attributes entailed multivariate Cox regression analysis. Subsequent corroboration of this signature in the validation cohort entailed the use of the aforementioned formula to quantitate the risk score in individual patients. The cutoff value employed in the training cohort was applied in validation cohort with the categorization of patients as high-risk and low-risk groups. Corroboration entailed both the Kaplan-Meier and the time-dependent ROC analyses.

### 2.4. The Predictive Nomogram

We further depicted nomograms built on the “rms” R package with the aforementioned lncRNA signature and other prognostic contributors for OS prediction in HNSCC patients (1 year, 3 years, and 5 years). We also computed the calibration curve to probe its accuracy.

### 2.5. Functional Enrichment Analysis

This entailed scrutiny of the Kyoto Encyclopedia of Genes and Genomes (KEGG) pathway analysis with Gene set enrichment analysis (GSEA) (versionv4.1.0, http://www.gsea-msigdb.org/gsea/downloads) in the risk groups employing our NETosis-related lncRNA signature.

### 2.6. All-Inclusive Probing of Immune Cell Profile and ICB Therapy in Both Risk Groups

The measure of tumor-infiltrating immune cells in HNSCC samples was probed employing CIBERSORT [27], CIBERSORT−ABS [27], QUANTISEQ [28], XCELL [29], MCPcounter [30], EPIC [31], and TIMER [32] algorithms. Both risk groups were scrutinized for NETosis and immune functioning by ssGSEA or single-sample GSEA employing the “GSVA” package, while literature was scored for plausible genes of immune checkpoint molecules. In order to gauge the impact of the signature in patient prognosis post-ICB therapy, ssGSEA was done with the gene set of NETosis employing that the “GSVA” package of R in two cohorts in which ICB therapy (anti-PD-L1/PD-1) was administered [33, 34] to get individual NETosis scores. These scores (median values) were utilized to group patients into high and low scores. The relevance of the signature to predict ICB therapy response entailed relevant survival analyses.

### 2.7. Chemotherapy Response with Our NETosis-Related lncRNA Signature

The response to chemotherapy in the patients was scored employing the R package “pRRophetic” [35].

### 2.8. Cell Culture

This work entailed the use of normal human immortalized nasopharyngeal epithelial cell line (NP69) and human nasopharyngeal carcinoma cell lines (CNE1, HNE1, TW03, and SUNE1). All cells were cultivated in RPMI-1640 medium (GIBCO) supplemented with 7% fetal bovine serum (ExCell Bio) in 5% CO_2_ at 37°C.

### 2.9. Quantitative Real-Time PCR

Total RNA of NP69, CNE1, HNE1, or TW03 cells was collected employing the RNA-Quick Purification kit (ESscience) adhering to the requisite and prescribed protocols. cDNA was synthesized employing the RNA reverse transcription kit (ESscience) as per the prescribed instructions. Real-time PCR amplification ensued with SYBR Green (Vazyme) and the following sets of primers: AC079336.5 (5′-CACAATCCCACGCTGTACCT-3′ and 5′-CAGGTGTCCTCAGAAAGCGT-3′), AL645933.2 (5′-GCTTGCTGACTCTGTGGACT-3′ and 5′-AGTTCAGGTCACCAGTCCCT-3′), LINC00426 (5′-TGCAGGCTTTGTAGACCCTC-3′ and 5′-TTGCGGGTGATTTACTGGGG-3′), LINC00623 (5′-AGCTTCTCTGCAGGTCACAC-3′ and 5′-TGGGCCACCCTTGAACATTT-3′), and GADPH (5′-CTGGGCTACACTGAGCACC-3′ and 5′-AAGTGGTCGTTGAGGGCAATG-3′). All samples were subjected to scrutiny in triplicate, and each target gene was normalized by GADPH. qPCR and analyses were performed using the LightCycler 480 Instrument (ROCHE) and software.

### 2.10. Colony Formation Assay

CNE1 or SUNE1 cells were placed in triplicate with 500 cells per well in 12-well plates (BIOFIL) and cultured in RPMI-1640 medium (GIBCO) supplemented with 7% fetal bovine serum (ExCell Bio) for 10 days. Then, the plates were washed twice with PBS and fixed with 75% alcohol for 1 hour. After washing twice with PBS, the cells were stained with crystal violet for 2 hours. Then, the crystal violet was washed off, and the number of colonies was counted.

### 2.11. Cell Proliferation Assay

MTT assay was used to assess the relative viability of the cells. Briefly, cells were seeded at 1000 cells per well in 96-well plates and cultured overnight in RPMI-1640 medium containing 7% FBS at 24 hours posttransfection, respectively. Add 10 *μ*L of MTT labeling reagent and continue incubation for 4-6 h. Read the spectrophotometry of the samples at 570 nm. Data were analyzed using GraphPad Prism 8 (GraphPad Software, La Jolla, CA, USA).

### 2.12. Wound Healing Assay

Cells were seeded into 6-well tissue culture plates at an appropriate density of 50-60% and cultured in RPMI-1640 medium (GIBCO) supplemented with 7% fetal bovine serum (ExCell Bio) for 24 hours before becoming a monolayer. A linear wound was scraped on the cell monolayer with a 20 *μ*L pipette tip. After scraping, the cells were washed off by gently rinsing the medium twice and then cultured in RPMI-1640 medium without fetal bovine serum. Wounds were imaged under a microscope at 0, 24, 48, and 72 hours. Three areas were randomly photographed.

### 2.13. Plasmids and Transfection

LINC00426 plasmids and control plasmids were purchased from Shanghai Genechem Co. Ltd. Plasmid transient transfection was performed using Lipofectamine 3,000 (Invitrogen) according to the manufacturer's instructions. And then, cells were collected for subsequent experiments after 24 hours of transfection.

### 2.14. Western Blotting

Whole-cell extracts were generated by direct lysis with 1× Cell Lysis Buffer (Cell Signaling Technology, #9873) with 1 mM phenylmethylsulphonyl fluoride (PMSF) added immediately before use. Samples with 6× SDS sample buffer added were heated at 100°C for 10 min and resolved by SDS-PAGE and then transferred to a PVDF membrane. The membranes were then examined with primary antibodies, followed by the corresponding HRP-conjugated anti-mouse or anti-rabbit (Proteintech) secondary antibodies. The following antibodies were used: *α*-tubulin (1 : 1000, Proteintech), cGAS (1 : 1000, Abcepta), TBK1 (1 : 1000, Proteintech), phospho-TBK1 (1 : 1000, CST), STING (1 : 1000, Proteintech), phospho-STING (1 : 1000,CST), IRF3 (1 : 1000, Proteintech), and phospho-IRF3 (1 : 1000, CST).

### 2.15. Statistical Analyses

The Wilcox test was employed to probe the relative amounts of immune checkpoint molecules and immune cells infiltrating the malignancy in both the risk groups. The lncRNA signature and its link with clinicopathological factors were probed by the chi-squared test. As elucidated above, the identification of the independent factors in OS prognosis entailed multivariate Cox regression analyses. The accuracy of prognosis prediction was gauged by ROC analyses. R software (Version 4.1.0) and SPSS (Version 23.0) were employed for all these computations.

## 3. Results

### 3.1. Patient Characteristics in Both Cohorts

Random assignment of HNSCC patients who met eligibility criteria (*n* = 499) was done into training (*N* = 333) and validation (*N* = 166) cohorts in a 2 : 1 ratio. The clinical characteristics and pathological records have been detailed in Table 1. The training cohort included 246 (73.9%) male and 87 (26.4%) female patients with 50.8% patients over 60 years old, while the validation cohort included 120 (72.3%) male and 46 (27.7%) female patients with 51.8% patients over 60 years old. A total of 204 (61.3%) patients and 110 (66.3%) patients had smoking history in the training cohort and validation cohort, respectively. Most of the patients had absence of HPV evaluation in both groups. There were 23 patients (6.9%) confirmed with positive HPV status and 53 patients (15.9%) confirmed with negative HPV status in the training cohort. Similarly, there were 10 patients (6.0%) confirmed with positive HPV status and 26 patients (15.7%) confirmed with negative HPV status in the validation cohort. For the training cohort, pathological evaluation showed that 236 (70.9%) patients were classified as moderate to poor differentiation grade (grade 1-2), and 84 (25.2%) patients were classified as well differentiation grade (grade 3-4). Besides, 17 (5.1%) patients, 45 (13.5%) patients, and 56 (16.8%) patients, and 170 (51.1%) were classified as TNM stages I, II, III, and IV HNSCC, respectively. For the validation cohort, pathological evaluation showed that 123 (74.1%) patients were classified as moderate to poor differentiation grade (grade 1-2), and 37 (22.3%) patients were classified as well differentiation grade (grade 3-4). Besides, 8 (4.8%) patients, 24 (14.5%) patients, and 22 (13.3%) patients and 89 (53.6%) were classified as TNM stage I, II, III, and IV HNSCC, respectively. Overall, no significant differences were detected in age, gender, smoking history, HPV status, tumor grade, and tumor stage between training and validation cohorts.

### 3.2. Data Collection and Identification of NETosis-Related lncRNAs

Firstly, we included the data of RNA-seq and clinical data of 528 HNSCC patients from TCGA; then, 44 normal samples and 1 sample lacked survival data were excluded (final patient number = 499). Then, 24 NETosis-linked genes were delineated in HNSCC patients as outlined above. The correlation between 564 NETosis-related lncRNAs and 24 NETosis-linked genes was evaluated by Pearson's correlation analysis, and the NETosis-related lncRNAs were identified according to the standard that the *P* value was less than 0.001 (*P* < 0.001), and the absolute value of Pearson's correlation coefficient was more than 0.4 (|*R*| > 0.4).

### 3.3. Building a Prognostic NETosis-Related lncRNA Signature in HNSCC Patients

**Figure 1:**
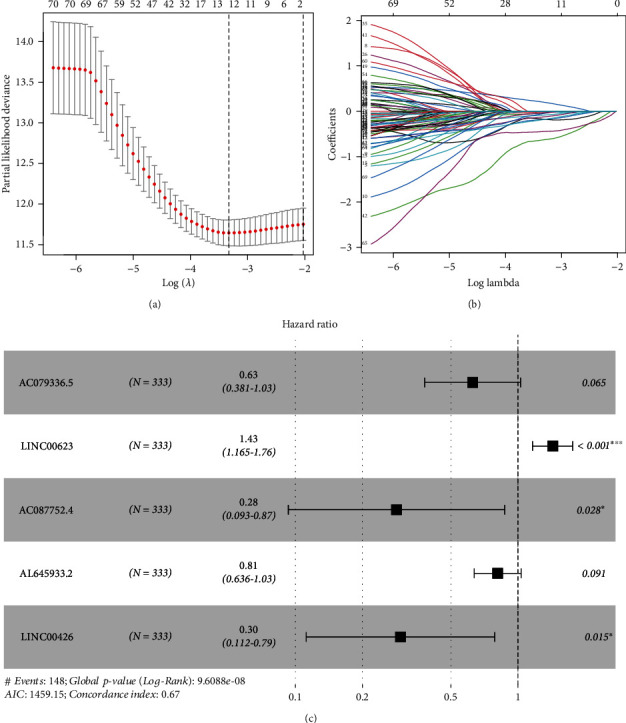
The construction of a prognostic model in head and neck squamous cell carcinoma (HNSCC) patients. (a) 12 NETosis-related lncRNAs were selected by the least absolute shrinkage and selection operator (LASSO) regression model according to minimum criteria. (b) The coefficient of NETosis-related lncRNAs was calculated by LASSO regression. (c) Forest plots showing the results of the multivariate Cox regression analysis between the 5 NETosis-related lncRNAs and overall survival (OS) of HNSCC.

**Figure 2:**
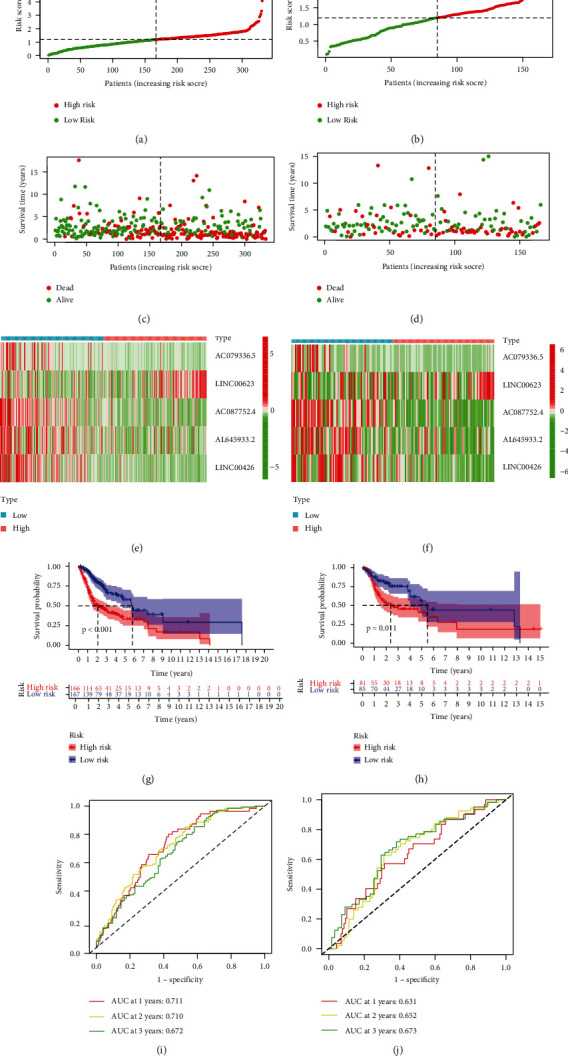
The prognostic performance of the NETosis-related lncRNA signature in the training cohort and validation cohort. (a)The distribution of the risk scores in the training cohort. (b) The distribution of the risk scores in the validation cohort. (c)The scatter plots showing whether the samples were alive or not in the training cohort. (d) The scatter plots showing whether the samples were alive or not in the validation cohort. (e) Heat map of the expression of 5 NETosis-related lncRNAs in the training cohort. (f) Heat map of the expression of 5 NETosis-related lncRNAs in the validation cohort. (g) Kaplan-Meier curves for the overall survival of patients in the high- and low-risk groups in the training cohort. (h) Kaplan-Meier curves for the overall survival of patients in the high- and low-risk groups in the validation cohort. (i) Area under time-dependent ROC curve (AUC) of time-dependent receiver operating characteristic (ROC) curves verified the prognostic accuracy of the risk score in the training cohort. (j) AUC of time-dependent ROC curves verified the prognostic accuracy of the risk score in the validation cohort.

A total of 499 HNSCC patients were randomly assigned to either training set or validation set. The initial univariate Cox regression analysis unveiled the prognostic lncRNAs in HNSCC patients based on training set. The overlapping prognostic lncRNAs and NETosis-related lncRNAs were identified as the candidate lncRNAs for the NETosis-related lncRNA signature, which resulted in 113 lncRNAs. In other words, these 113 lncRNAs were significantly associated not only with NETosis but also with prognosis of HNSCC patients. Subsequent LASSO Cox regression to reduce the multicollinearity unearthed 12 lncRNAs (Figures 1(a) and 1(b)). Ensuing multivariate Cox regression analysis ultimately highlighted five NETosis-related lncRNAs as optimal prognostic factors in HNSCC patients (Figure 1(c)). The lncRNA signature (AC079336.5, LINC00623, AC087752.4, AL645933.2, and LINC00426) was unveiled employing the least AIC score (Table 2). The computation of the risk score based on the signature was as per the following formula: risk score = −0.468 × AC079336.5 + 0.360 × LINC00623–1.257 × AC087752.4–0.209 × AL645933.2–1.215 × LINC00426. Then, each patient in the training set got a risk score based on the formula. The grading of patients in training set was done as high-risk (*n* = 166) and low-risk (*n* = 167) groups with the median risk score value. No evident differences between both risk groups emerged for age, gender, smoking status, and tumor stage, while HPV positive and grade 3-4 were more common in the low-risk group (*P* < 0.001 and *P* = 0.020, respectively) (Table 3). The survival outcome, risk status, and expression profile of lncRNAs of each patient are documented in Figures 2(a), 2(c), and 2(e), respectively, with the high-risk patients documenting a lower probability of survival vs. the low-risk patients. Further, the OS was shortened in the high-risk patients as evidenced by the Kaplan-Meier method (Figure 2(g), *P* < 0.001). The signature documented significant predictive roles regarding the 1-year OS, 2-year OS, and 3-years OS with the AUC of the ROC analyses at 0.711, 0.710, and 0.672, respectively (Figure 2(i)).

### 3.4. Corroboration of the lncRNA Signature in the Validation Cohort

To verify the accuracy of the NETosis-related lncRNA signature, the computation of the risk score of validation cohort entailed the one employed in the training cohort. On the same lines, the categorization of the validation group patients ensued as high-risk (*N* = 81) and low-risk (*N* = 85) groups (Table 3) employing the aforementioned cutoff value. As shown in Table 3, both groups documented no conspicuous differences for age, gender, smoking status, and tumor grade, while HPV positive and stage I were more common in the low-risk patients (*P* = 0.034 and *P* < 0.001, respectively). The survival outcome, risk status, and lncRNA profile are illustrated in Figures 2(b), 2(d), and 2(f), respectively. On similar lines, the low-risk patient group was demonstrative of an elevated survival vs. the high-risk patients with the latter group demonstrative of a diminished OS as evidenced by the Kaplan-Meier method (Figure 2(h), *P* = 0.011). The signature documented significant predictive roles in the 1-year OS, 2-years OS, and 3-years OS with the AUC at 0.631, 0.652, and 0.673, respectively (Figure 2(j)).

### 3.5. The Independent Functioning of the lncRNA Signature for HNSCC Prognosis

**Figure 3:**
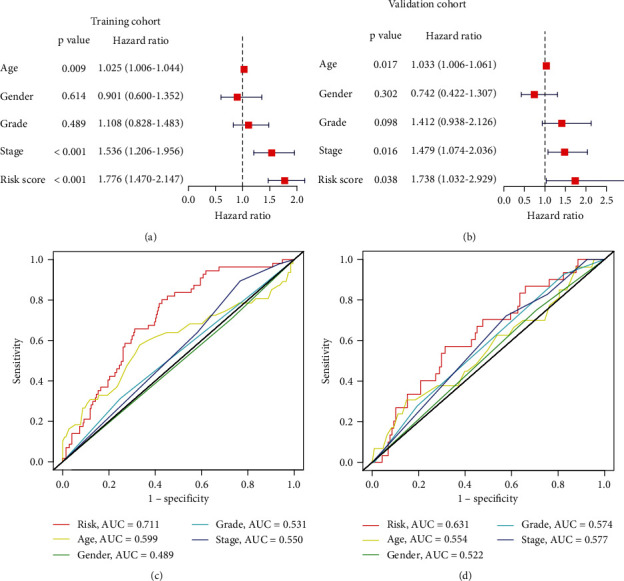
Independent prognostic value of the NETosis-related lncRNA signature in the training cohort and validation cohort. (a) Results of the multivariate Cox regression analysis regarding OS in the training cohort. (b) Results of the multivariate Cox regression analysis regarding OS in the validation cohort. (c) Area under time-dependent ROC curve (AUC) of receiver operating characteristic (ROC) curves compared to the prognostic accuracy of the risk score and other clinicopathological in the training cohort. (d) AUC of ROC curves compared to the prognostic accuracy of the risk score and other clinicopathological in the validation cohort.

Multivariate Cox regression facilitated the ascertaining of our lncRNA signature as an independent factor in HNSCC prognosis (training cohort: HR = 1.776, 95%CI = 1.470–2.147, *P* < 0.001; validation cohort: HR = 1.738, 95%CI = 1.032–2.929, *P* = 0.038, respectively) (Figures 3(a) and 3(b)). The ROC curve analysis probing its specificity and sensitivity documented the strength of the signature with an AUC of 0.711 and 0.631 for the training and validation cohorts, respectively, exceeding that of the remaining factors probed (Figures 3(c) and 3(d)). Thus, our NETosis-related lncRNA signature could function as an independent tool for prognosis prediction of HNSCC patients.

### 3.6. The Predictive Nomogram: Development and Corroboration

**Figure 4:**
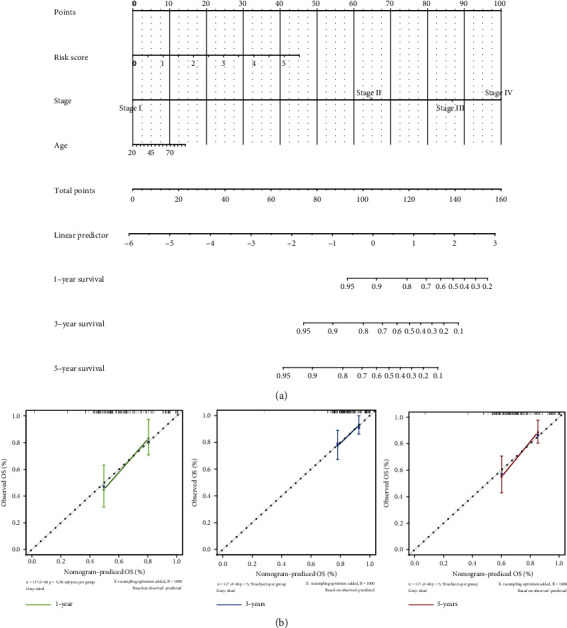
Building and validation of the nomogram to predict the overall survival of patients. (a) Nomogram plot was built based on risk score, age, and stage in the whole cohort. (b) Calibration curve of the nomogram.

To provide a useful prediction model for survival probability of HNSCC patients, a nomogram including clinical features and risk score was constructed. As the multivariate Cox regression analysis indicated the clinical feature including stage, age, and risk score as independent factors, the nomogram was constructed employing the stage, age, and signature (Figure 4(a)).The prediction of the OS (1 year, 3 years, and 5 years) entailed the construction of a prognostic nomogram encompassing all the independent factors discerned to depict a risk gauging system comprehensively and visually. The gauging of the OS employing this nonogram via calibration curves was illustrated in Figure 4(b). A conspicuous agreement emerged for the OS predicted by the nonogram and the authentic values across various follow-up periods. The stability and accuracy of our nomogram encompassing our lncRNA signature with clinical features can predict the outcome of individual patients, thus bringing benefits to clinicians and patients.

### 3.7. GSEA for Vital Pathway Scoring

**Figure 5:**
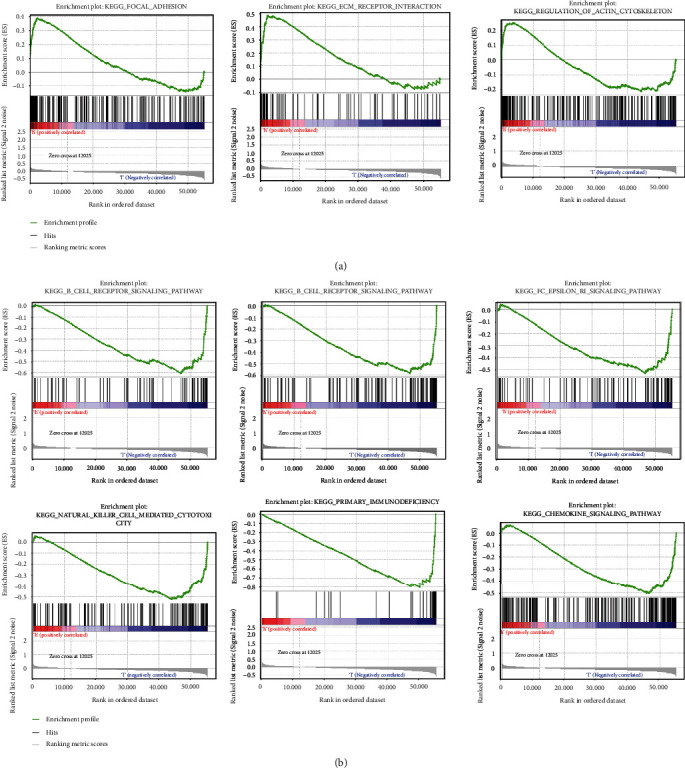
Gene set enrichment analysis (GSEA) of the two groups based on the NETosis-related lncRNA prognostic signature. (a) GSEA results show significant enrichment of glucose and protein metabolism pathways in the high-risk head and neck squamous cell carcinoma (HNSCC) patients. (b) GSEA results show significant enrichment of immunoregulatory pathways against cancer in the low-risk HNSCC patients.

To explore the potential signal pathways or functions of NETosis-related lncRNAs in HNSCC, we applied gene set enrichment analysis (GSEA) to two cohorts. As elucidated above, this entailed scoring both groups for pathways documenting variations by KEGG analysis employing GSEA. An upregulation emerged for genes in focal adhesion, ECM receptor interaction, and actin cytoskeleton regulation in the high-risk patient set (Figure 5(a)). The low-risk dataset documented a conspicuous upregulation for anticancer immune pathways inclusive of B cell receptor, T cell receptor and Fc*ε*RI signaling, natural killer cell-mediated cytotoxicity, and primary immunodeficiency along with the chemokine signaling pathway (Figure 5(b)).The results of the KEGG of NETosis-related lncRNAs suggested that high-risk patient set was more possibly to exhibit tumor metastasis and worse prognosis, while the upregulation of anticancer immune pathways in low-risk patient set indicated an immune status unfavorable to tumor growth and better prognosis.

### 3.8. ICB Therapy Outcome Determined by the Immune and NETosis Status across Both Risk Groups

**Figure 6:**
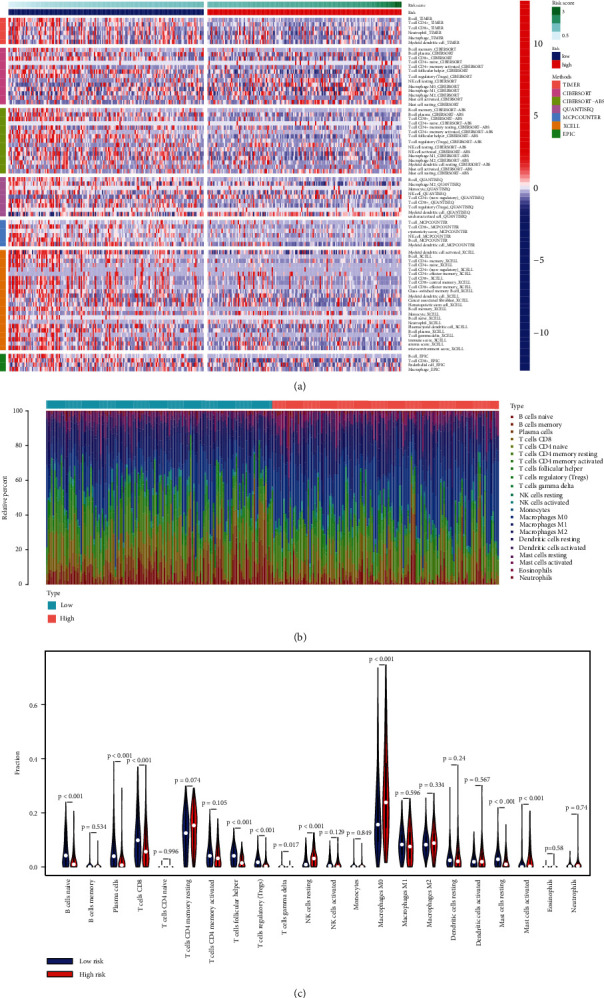
The immune cell infiltration landscape in head and neck squamous cell carcinoma (HNSCC). (a) Heat map for immune cell infiltration landscape based on the CIBERSORT, CIBERSORT−ABS, QUANTISEQ, XCELL, MCPcounter, EPIC, and TIMER algorithms among high- and low-risk groups. Only items with significant differences will be displayed; *P* value < 0.05 was controlled. (b) Barplot of the tumor-infiltrating cell proportions based on CIBERSORT algorithm. (c) Violin plot showed the different proportions of tumor-infiltrating cells between different groups based on CIBERSORT algorithm.

To investigate the relationship between NETosis-related lncRNAs and immune status, the various algorithms outlined mentioned in the materials section were employed to probe the immune cells and pathways in both the risk groups, which showed significant difference for proportions of different tumor-infiltrating immune cells between the low-risk and high-risk groups (Figure 6(a)). Further, CIBERSORT facilitated the immune cell infiltration to be gauged. As shown in Figures 6(b) and 6(c), the high-risk group documented an evident diminishing of naive B cell, plasma cells, CD8+ T cell, follicular helper T cells, regulatory T cells, gamma and delta T cells, and resting and activated mast cells vs. that in the low-risk patient set; however, the proportion of resting NK cells and M0 macrophages was significantly higher in high-risk group.

**Figure 7:**
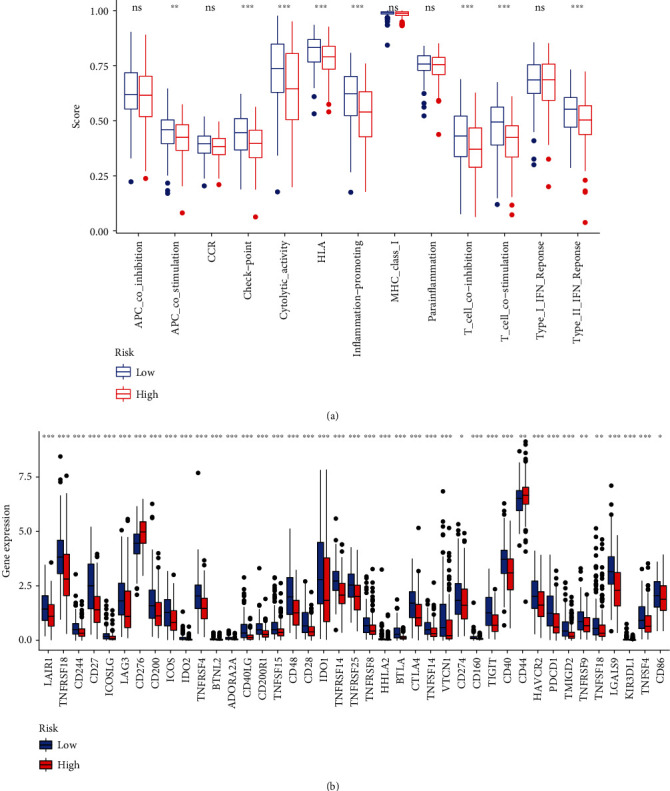
Barplot shows that the low-risk group and high-risk groups exhibit different immune statuses. (a) Single-sample gene set enrichment analysis (ssGSEA) for the immune functions between high and low head and neck squamous cell carcinoma (HNSCC) risk groups. (b) The expression levels of immune checkpoints between high and low HNSCC risk groups (^∗^*P* < 0.05, ^∗∗^*P* < 0.01, and ^∗∗∗^*P* < 0.001).

Then, the difference in immune functions between the two groups was compared. Both groups were demonstrative of evident variations in the ssGSEA for T cell functions like checkpoint (inhibition), cytolytic activity, HLA, inflammation status, T cell coinhibition, and T cell costimulation, which is indicative of the low-risk group documenting an elevated T cell functions (Figure 7(a)). Based on the above considerations, the low-risk cohort can be assigned plausibly as a “hot tumor” demonstrative of elevated immune checkpoint (inhibition) as per the augmented immune cell infiltration and immune responses. Our prognostic signature is demonstrative of an augmented effects in the low-risk group by ICB therapy. The immune checkpoint molecule profiles were then scored in both groups. We found that the low-risk group documented an elevated level of PDL1 (CD274), CTLA4, IDO1, and LAG3 documented vs. the high-risk patient group (Figure 7(b)).

**Figure 8:**
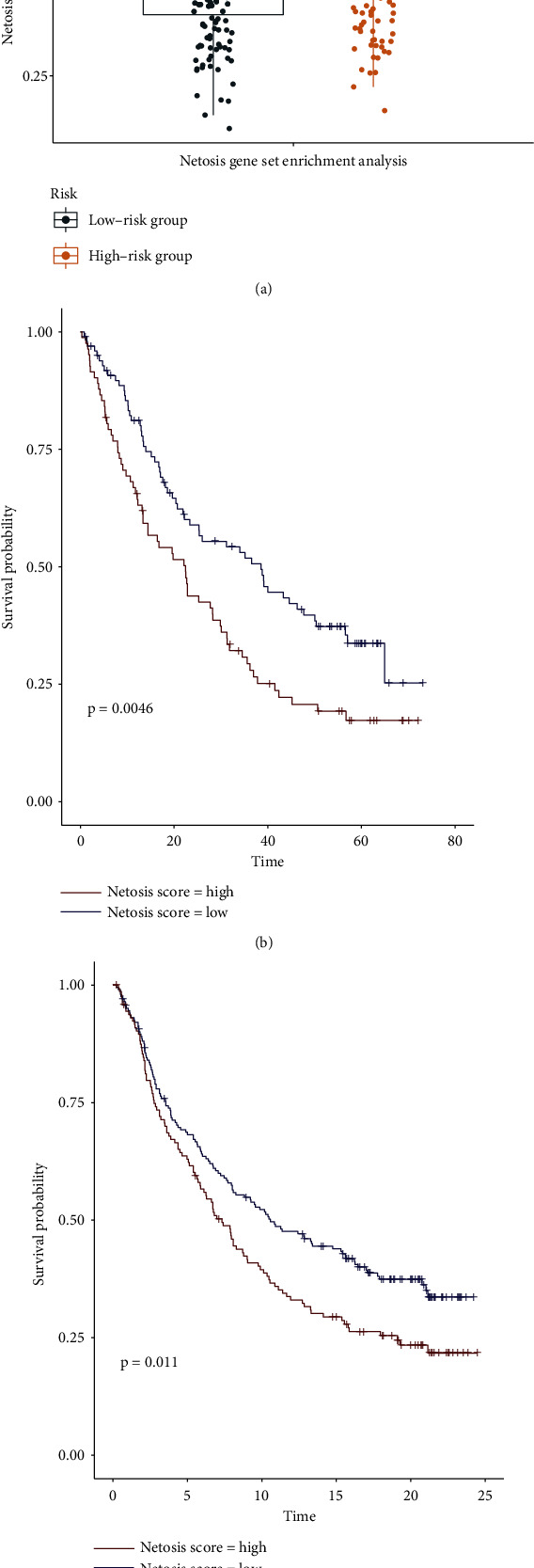
The prognostic value of NETosis score in patients with immunotherapy. (a) ssGSEA was used to calculate the level of NETosis between the high-risk and low-risk group. (b) Kaplan-Meier curves for the overall survival of patients in the David A. Braun et al.'s clear cell renal cell carcinoma cohort. (c) Kaplan-Meier curves for the overall survival of patients in the Sanjeev Mariathasan et al.'s urothelial cancer cohort.

To further explore the prognostic value of NETosis score in patients with immunotherapy, firstly, we confirmed that patients in the high-risk group of HNSCC have higher NETosis score by using the “GSVA” package, which revealed activation of NETosis in the high-risk group vs. the low-risk group (Figure 8(a)). Then, we performed ssGSEA by using the NETosis gene panel in two cohorts administered with immunotherapy employing the “GSVA” package as documented in Materials and Methods to compute the NETosis score of individual samples. Following the categorization of samples as high and low scores employing the median score value, the patients documented a better survival profile when the NETosis score was lowered (Figures 8(b) and 8(c)). This suggests that the low-risk group based on our NETosis-related lncRNA signature has a lower NETosis score and better survival after receiving ICB therapy. These documented outcomes are also corroborative of the plausible impact of our NETosis-related lncRNA signature to predict how fitting ICB would be in these patients.

To summarize, a link between the immune cell status and NETosis score with this lncRNA signature emerged with the high-risk group possibly documenting a diminishing of immune cell infiltration and activity with downregulation of immune checkpoint molecules with lowered survival post-ICB therapy as opposed to the lower-risk cohort. NETosis-related lncRNAs-NETosis-antitumor immunity may be a signaling cascade, which may pave the way for a future novel therapeutic approach to target the malignancy in HNSCC patients.

### 3.9. Scoring the Chemotherapy Response with the NETosis-Related lncRNA Signature

**Figure 9:**
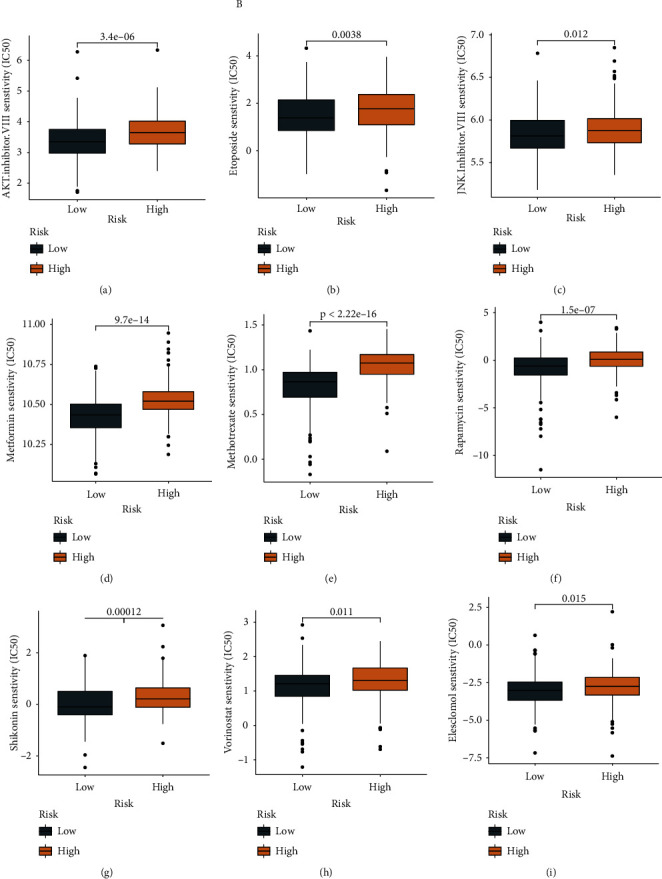
Evaluation of chemosensitivity by the risk model. The model showed that low-risk scores were associated with a lower half inhibitory centration (IC_50_) for chemotherapeutics such as (a) AKT inhibitor, (b) etoposide, (c) JNK inhibitor V, (d) metformin, (e) methotrexate, (f) rapamycin, (g) shikonin, (h) vorinostat, and (i) elesclomol.

To further probe the value of our lncRNA signature in patients undergoing varying chemotherapy regimens, the “pRRophetic” approach was employed to predict the chemotherapy response in both risk groups. A diminished estimated IC_50_ was documented by the low-risk patients vs. the high-risk ones in terms of these chemotherapy drugs: AKT inhibitor VIII, etoposide, JNK inhibitor VIII, metformin, methotrexate, rapamycin, shikonin, vorinostat, and elesclomol (Figures 9(a)–9(i)) (*P* < 0.05). The results showed that patients in the high-risk group had poorer outcomes vs. in the low-risk patients when receiving the above chemotherapy regimens.

### 3.10. lncRNA Expression from Our NETosis-Related lncRNA Signature In Vitro

**Figure 10:**
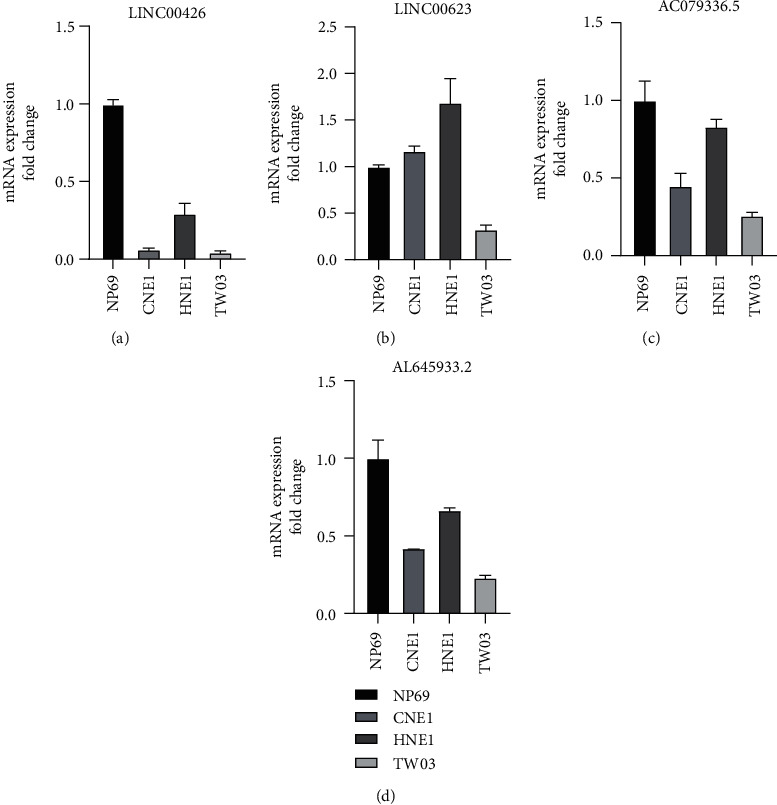
lncRNA expression from our NETosis-related lncRNA signature. (a) mRNA expression of LINC00426 in different cell lines. (b) mRNA expression of LINC00623 in different cell lines. (c) mRNA expression of AC079336.5 in different cell lines. (d) mRNA expression of AL645933.2 in different cell lines.

There are five lncRNAs in our prognostic model, of which four are protective factors and one is a risk factor. The high-risk group documented an elevated expression of the risk factor with a diminished expression of protective factors. This led to whether tumor cell lines also document this similar expression as the high-risk group. We compared immortalized nasopharyngeal epithelial cell lines (NP69) and human nasopharyngeal carcinoma cell lines (CNE1, HNE1, and TW03). As we described above, protective factors include AC079336.5, AC087752.4, AL645933.2, and LINC00426, while LINC00623 is a risk factor. qPCR revealed lowered expression of LINC00426 in CNE1, HNE1, and TW03 than in NP69 and lowered expression of AC079336.5 and AL645933.2 in CNE1 and TW03 than in NP69, while the expression level of LINC00623 is inconsistent among control cell and tumor cell lines (Figures 10(a)–10(d)). The above results reflect the reliability of our model to a certain extent.

### 3.11. Verification the Effect of lncRNA on Proliferation and Migration In Vitro

To further investigate the role of LINC00426, we tested the effect of LINC00426 on the proliferation and migration of human nasopharyngeal carcinoma cell lines by transient transfection of overexpressing plasmids. The expression of mRNA was assessed by qPCR. We then investigated whether the cell proliferation and migration were inhibited upon LINC00426 overexpression in the nasopharyngeal carcinoma cell lines cells (CNE1 and SUNE1). However, cell proliferation assays showed that overexpression of LINC00426 did not affect cell viability compared to the negative group (Figure S2A and Figure S2C). There was also no change in colony formation ability after overexpression of LINC00426 in CNE1 and SUNE1 cells (Figure S2B and Figure S2D). In addition, the wound healing assay also showed that overexpression of LINC00426 did not affect the migration ability of CNE1 and SUNE1 cells (Figure S2E and Figure S2F).

### 3.12. LINC00426 Contributes to the STING Signaling Pathway

**Figure 11:**
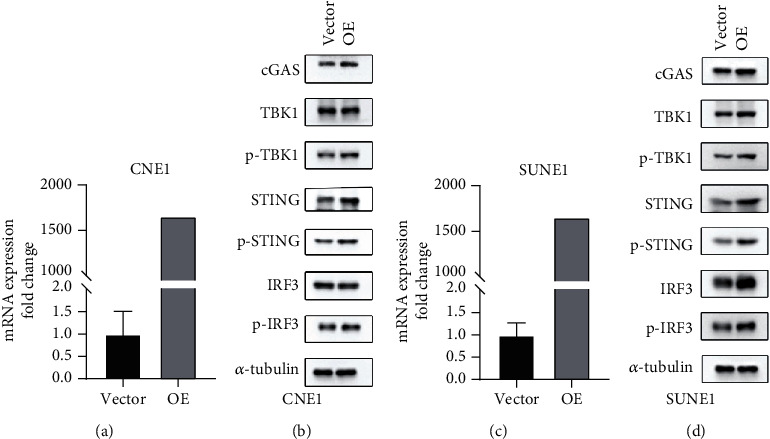
Changes in STING signaling pathway-related proteins in different cell lines transfected with or without LINC00426 overexpression plasmids. (a) mRNA expression of LINC00426 in CNE1 cells. (b) Western blot for cGAS, TBK1, STING, and IRF3 in CNE1 cells. (c) mRNA expression of LINC00426 in SUNE1 cells. (d) Western blot for cGAS, TBK1, STING, and IRF3 in SUNE1 cells.

The prognostic model of lncRNA that we built was able to not only predict the prognosis of patients but also identify “hot” and “cold” tumors. Therefore, we try to explore the possibility of lncRNA regulation of immunity in vitro. We hypothesized that LINC00426 regulated immune cell infiltration; we overexpressed LINC00426 in CNE1 and SUNE1 cells (Figures 11(a) and 11(c)) and detected the expression of cGAS-STING-TBK1-IRF3 signaling pathway. The data exhibited that LINC00426 overexpressed significantly enhanced p-STING, p-TBK1, and p-IRF3 protein levels in both CNE1 and SUNE1 cells (Figures 11(b) and 11(d)). The activation of the STING signaling pathway is known to further promote the secretion of cytokines such as CXCL10, CCL5, ISG15, and ISG56, thereby recruiting B cells, T cells, and promoting immune cell infiltration. These data explain to some extent the role of our prognostic model in predicting “hot” and “cold” tumors.

## 4. Discussion

To our knowledge, our study was the first to probe a NETosis-related lncRNA signature to predict HSNCC prognosis and group a patient set into high-risk and low-risk groups. An evident increase in anticancer immune pathways was documented in the low-risk group by functional enrichment analysis. In the meanwhile, a close association emerged for our lncRNA signature with the immune cell infiltration and NETosis profiles in HNSCC. To expound, an immunologically “cold” profile emerged in the high-risk group, which included diminished immune cell infiltration and activity, and dampened immune checkpoint molecule expression, while an immunologically “hot” profile emerged in the low-risk group. The putative potential of our signature was also corroborated in the two patient sets who received ICB as evidenced in the ssGSEA to predict the relevance of ICB in patients. The effective prediction of the response to select chemotherapy drugs by the signature was also documented in HNSCC patients.

The involvement of lncRNAs in NETosis has been demonstrated in several studies. For example, Li et al. reported that an upregulation of IL-12A due to lncRNA X-inactive specific transcript by binding to miR-21 stimulates NETosis and accelerates primary graft dysfunction subsequent to lung transplantation [36]. Gao and Zhang documented that diminishing of lncRNA MINCR inhibits NETosis and is involved in LPS-evoked acute injury and inflammatory response [37]. Nonetheless, there are few studies on elucidating lncRNAs connected with NETosis in oncogenesis and moreover HNSCC. There were many researches focusing on figuring out NETosis-associated gene; we employed Pearson's correlation analysis on these genes and lncRNAs to identify NETosis-related lncRNAs, which initially resulted in 113 NETosis-associated lncRNAs that regarded to be associated with the survival of HNSCC patients by univariate Cox regression. Further analyses narrowed down on five NETosis-related lncRNAs: AC079336.5, LINC00623, AC087752.4, AL645933.2, and LINC00426. Of these 5 lncRNAs in our prognostic signature, the involvement of LINC00426 in oncogenesis has been documented. For example, the regulation of miR-455-5p by LINC00426 to boost lung adenocarcinoma progression was demonstrated [38], while LINC00426 was downregulated in non-small-cell lung cancer patient tumor tissues and correlated with poor prognosis [39]. Another study documented that LINC00426 contributes to doxorubicin resistance by sponging miR-4319 in osteosarcoma [40]. Our results showed that LINC00426 overexpressed upregulated STING signaling pathway in HNSCC cell lines, which indicated that innate immunity was activated [41]. Our data explain to some extent the role of our prognostic model in predicting “hot” and “cold” tumors, which illustrated that our model is reliable. For the four remaining NET-related lncRNAs (AC079336.5, LINC00623, AC087752.4, and AL645933.2), research on their involvement in cancer has not yet been documented. We are not able to verify the function of the other four lncRNAs within severe constraints of time and money.

The involvement of NETosis in tumorigenesis and therapeutic approaches is being documented in several reports. The definition of NETosis entailed NET release and cell death involving ROS specifically in cells of hematopoietic origin [8]. Several signaling cascades are then stimulated by this NETosis production in tumors encompassing the malignancy itself with blood cells like leukocytes and platelets and establish an inflammatory microenvironment to boost tumor progression [42]. As outlined above, the involvement of this process and NETosis-related lncRNAs in the HNSCC immune microenvironment warrants scrutiny. This work documented a diminishing of crucial immune pathways involved in antitumor functioning like natural killer cell cytotoxicity, B cell/T cell receptor signaling, and elevated NETosis in the high-risk group in the relevant assays. This was suggestive of the plausible link between antitumor immunity and NETosis in HNSCC. Zhang et al. demonstrated the recruitment of neutrophils to trigger NETosis by IL17 to exclude cytotoxic CD8 T cells in pancreatic ductal adenocarcinoma. Interestingly, NET inhibition was documented in a recipient animal model with an arginine deiminase 4 gene (the enzyme PAD4, vitally involved in mediating NETosis from neutrophils) deletion with a better response to ICB in these murine systems emerging vs. those who expressed PAD4 and demonstrated NETs in the tumor microenvironment [43]. Another study has uncovered the inhibition of immune cytotoxicity by NETs via immune cell-target cell contact impairment and inhibition of NETosis by pharmacologically suppressing PAD4 augments tumor sensitivity to PD-1 + CTLA-4 dual checkpoint blockade in a syngeneic mouse model of breast cancer [9]. These results revealed a strong association between NETosis and antitumor immunity, which was consistent with our results.

In order to prove the hitherto unknown aspects of immune cell infiltration and NETosis in HNSCC, the former was scrutinized employing the algorithms listed earlier. A conspicuous diminishing of infiltration of cytotoxic cells inclusive of naive B cells and CD8+ T cells emerged in the high-risk patients vs. that of the low-risk patient set. The high-risk group also was demonstrative of diminished immune checkpoint molecule expression to be hence tagged as immunologically “cold” tumors to plausibly limit the response of ICB therapy as documented by our lncRNA signature. To corroborate this possibility, functional enrichment analysis was conducted, which revealed that anticancer immune pathways were significantly upregulated in the low-risk HNSCC group. Furthermore, probing of the cohorts with our NETosis-associated lncRNA signature unearthed an augmented survival post-ICB therapy in low-risk patients. These observations were indicative of the putative impact of our NETosis-related lncRNA signature to predict ICB response in patients to further guide treatments in the future.

An augmentation of NETosis emerged in the high-risk cohort as scrutinized by the “GSVA” package-based score. This leads us to hypothesize an augmented response to ICB therapy in this group by boosting immune cell infiltration by plausibly suppressing this NETosis in this high-risk group. The role of NETosis in anti-tumor immunity is being unearthed by ongoing work. Inhibition of NETosis is closely associated with antitumor immunity. Our research has provided the theoretical basis that high-risk HNSCC patients may benefit from the combination of ICB with NETosis inhibitors, which inhibit cell NETosis and increase immune cell infiltration to enhance the response to ICB therapy. This gains support with ongoing trials exploring the efficacy of concurrent NETosis inhibitors with other therapeutic strategies. The suitability of the neutrophil/NET system and the CXCR1/2 and the IL-8 pathway is receiving the center stage as therapeutic targets given their crucial importance. Concurrent administration of ICB with some CXCR1 and 2 inhibitors has been subjected to clinical testing. For instance, a phase I study is probing a combination of SX-682 (a CXCR1/2 inhibitor) and nivolumab (anti-PD1) in metastatic pancreatic ductal adenocarcinoma (NCT04477343). The concurrent administration of pembrolizumab (anti-PD1) with navarixin (a CXCR1/2 inhibitor) in advanced/metastatic solid tumors is being probed in a phase II study (NCT03473925) [42]. Our study may help provide clues to identify high-risk patients who may benefit more from the combination of ICB and NETosis inhibitors.

This work encompasses a few limitations. More in vivo or in vitro basic experiments are warranted to corroborate the potential molecular mechanistic aspects of NETosis-related lncRNAs in prognosis. In addition, clinical trials are urgently required to confirm whether inhibiting NETosis could improve the efficacy of immunotherapy in human HNSCC patients.

In conclusion, we identified the suitability of a NETosis-based lncRNA signature in the prognosis of HNSCC patients. Further variation in the immune cell profile and immune checkpoint molecule expression between the high-risk and low-risk groups are also documented. Our study suggests that NETosis inhibition may emerge as a strategy to augment the efficacy of immunotherapy in HNSCC patients.

## Figures and Tables

**Table 1:** Clinical characteristics of patients in the training cohort and validation cohort.

	Training cohort		Validation cohort		*P* value
	*N* = 333		*N* = 166		
	No.	%	No.	%	
Age					
≤60	164	49.2	80	48.2	0.824
>60	169	50.8	86	51.8	
Gender					
Female	87	26.4	46	27.7	0.706
Male	246	73.9	120	72.3	
Smoking					
Former and current smoker	204	61.3	110	66.3	0.276
Nonsmoker	129	38.7	56	33.7	
HPV status					
Negative	53	15.9	26	15.7	0.926
Positive	23	6.9	10	6.0	
Unknown	257	77.2	130	78.3	
Grade					
G1-2	236	70.9	123	74.1	0.749
G3-4	84	25.2	37	22.3	
Unknown	13	3.9	6	3.6	
Stage					
I	17	5.1	8	4.8	0.888
II	45	13.5	24	14.5	
III	56	16.8	22	13.3	
IV	170	51.1	89	53.6	
Unknown	45	13.5	23	13.9	

**Table 2:** Signature was identified based on the lowest Akaike information criterion (AIC).

Model	Prognostic signature combination	AIC
1	AC093278.2 + AC114730.3 + AC015911.3 + AC079336.5 + AC087392.5 + LINC00623 + RAB11B-AS1+ AL359881.1 + AC087752.4 + AL359921.1 + AL645933.2 + LINC00426	1468.15
2	AC093278.2 + AC015911.3 + AC079336.5 + AC087392.5 + LINC00623 + RAB11B-AS1 + AL359881.1 + AC087752.4 + AL359921.1 + AL645933.2 + LINC00426	1466.15
3	AC093278.2 + AC079336.5 + AC087392.5 + LINC00623 + RAB11B-AS1 + AL359881.1 + AC087752.4 + AL359921.1 + AL645933.2 + LINC00426	1464.52
4	AC093278.2 + AC079336.5 + AC087392.5 + LINC00623 + RAB11B-AS1 + AC087752.4 + AL359921.1 + AL645933.2 + LINC00426	1463.14
5	AC079336.5 + AC087392.5 + LINC00623 + RAB11B-AS1 + AC087752.4 + AL359921.1 + AL645933.2 + LINC00426	1461.83
6	AC079336.5 + LINC00623 + RAB11B-AS1 + AC087752.4 + AL359921.1 + AL645933.2 + LINC00426	1460.50
7	AC079336.5 + LINC00623 + AC087752.4 + AL359921.1 + AL645933.2 + LINC00426	1459.89
8	AC079336.5 + LINC00623 + AC087752.4 + AL645933.2 + LINC00426	1459.15

**Table 3:** Association between signature and clinicopathological manifestations.

	Training cohort (*N* = 333)		*P*	Validation cohort (*N* = 166)		*P*
	High risk	Low risk		High risk	Low risk	
	*n* = 166	*n* = 167		*n* = 81	*n* = 85	
Age (%)						
≤60	76 (45.8)	88 (52.7)	0.207	35 (43.2)	45 (52.9)	0.210
>60	90 (54.2)	79 (47.3)		46 (56.8)	40 (47.1)	
Gender (%)						
Female	46 (27.7)	41 (24.6)	0.512	26 (32.1)	20 (23.5)	0.218
Male	120 (72.3)	126 (75.4)		55 (67.9)	65 (76.5)	
Smoking (%)						
Former and current smoker	96 (57.8)	108 (64.7)	0.200	55 (67.9)	55 (64.7)	0.663
Nonsmoker	70 (42.2)	59 (35.3)		26 (32.1)	30 (35.3)	
HPV status (%)						
Negative	24 (14.5)	29 (17.4)	<0.001	12 (14.8)	14 (16.5)	0.034
Positive	1 (0.6)	22 (13.2)		1 (1.2)	14 (16.5)	
Unknown	141 (84.9)	116 (69.5)		68 (84.0)	62 (72.9)	
Grade (%)						
G1-2	129 (77.7)	107 (64.1)	0.020	64 (70.9)	59 (69.4)	0.178
G3-4	33 (19.9)	51 (30.5)		16 (19.8)	21 (24.7)	
Unknown	4 (2.4)	9 (5.4)		1 (1.2)	5 (5.9)	
Stage (%)						
I	5 (3.0)	12 (7.2)	0.261	7 (8.6)	1 (1.2)	<0.001
II	20 (12.0)	25 (15.0)		10 (12.3)	14 (16.5)	
III	33 (19.9)	23 (13.8)		13 (16.0)	9 (10.6)	
IV	86 (51.8)	84 (50.3)		48 (59.3)	41 (48.2)	
Unknown	22 (13.3)	23 (13.8)		3 (3.7)	20 (23.5)	

## Data Availability

The RNA sequencing data and patient characteristics of HNSCC patients were sourced from the TCGA database (https://portal.gdc.cancer.gov/repository). lncRNA and protein-coding gene annotations were ensued in the Ensembl human genome browser GRCh38.p13 (http://asia.ensembl.org/index.html). Gene set enrichment analysis (GSEA) was performed by GSEA software (versionv4.1.0, http://www.gsea-msigdb.org/gsea/downloads).

## Abbreviations

AIC: Akaike information criterion
AUC: Area under time-dependent receiver operating characteristic curve
CYT: Cytolytic activity
GSEA: Gene set enrichment analysis
HNSCC: Head and neck squamous cell carcinoma
HPV: Human papillomavirus
ICB: Immune checkpoint blockade
KEGG: Kyoto Encyclopedia of Genes and Genomes
LASSO: Least absolute shrinkage and selection operator
lncRNAs: Long noncoding RNAs
NETs: Neutrophil extracellular traps
NK: Natural killer
OS: Overall survival
ROC: Receiver operating characteristic
TCGA: The Cancer Genome Atlas.

## Supplementary Materials

Supplementary 1 Table S1: 24 NETosis-associated genes were identified from the literature.

Supplementary 2 Figure S1: the flow chart of our study. Figure S2: the effect of LINC00426 on nasopharyngeal carcinoma cell lines. (A) Cell proliferation assays showed that LINC00426 overexpression did not affect the viability of CNE1 cells. (B) Plate colony formation assay showed that LINC00426 overexpression did not affect the ability of colony formation in CNE1 cells. (C) Cell proliferation assays showed that LINC00426 overexpression did not affect the viability of SUNE1 cells. (D) Plate colony formation assay showed that LINC00426 overexpression did not affect the ability of colony formation in SUNE1 cells. Wound healing assay showed that LINC00426 overexpression did not affect the ability of migration both in CNE1 (E) and SUNE1 (F) cells.
