# Supplementary material for: Predicting the Immune Microenvironment and Prognosis with a NETosis-Related lncRNA Signature in Head and Neck Squamous Cell Carcinoma
Source: Biomed Res Int. 2022 Sep 12;2022:3191474. doi: 10.1155/2022/3191474 (PMC9485711; doi:10.1155/2022/3191474)
Supplement: Supplementary 1 — Table S1: 24 NETosis-associated genes were identified from the literature. [file 3191474.f1.docx]

**Table S1. 24 NETosis-associated genes were identified from the literature**

| **NETosis-Related Genes** |
| --- |
| MYD88, TLR2, PAD4, PRKCA, PKCB, PRKCZ, NOX3, NOX4, NOX1, CTSG, PRTN3, ELANE, MPO, GSDMD, IL1B , CXCL1, PLA2G7, CXCL8, CDK6, HMGB1, MMP9, AGER, CSF3, TGFB1 |
