# Supplementary figures and images for: Predicting the Immune Microenvironment and Prognosis with a NETosis-Related lncRNA Signature in Head and Neck Squamous Cell Carcinoma
Source: Biomed Res Int. 2022 Sep 12;2022:3191474. doi: 10.1155/2022/3191474 (PMC9485711; doi:10.1155/2022/3191474)

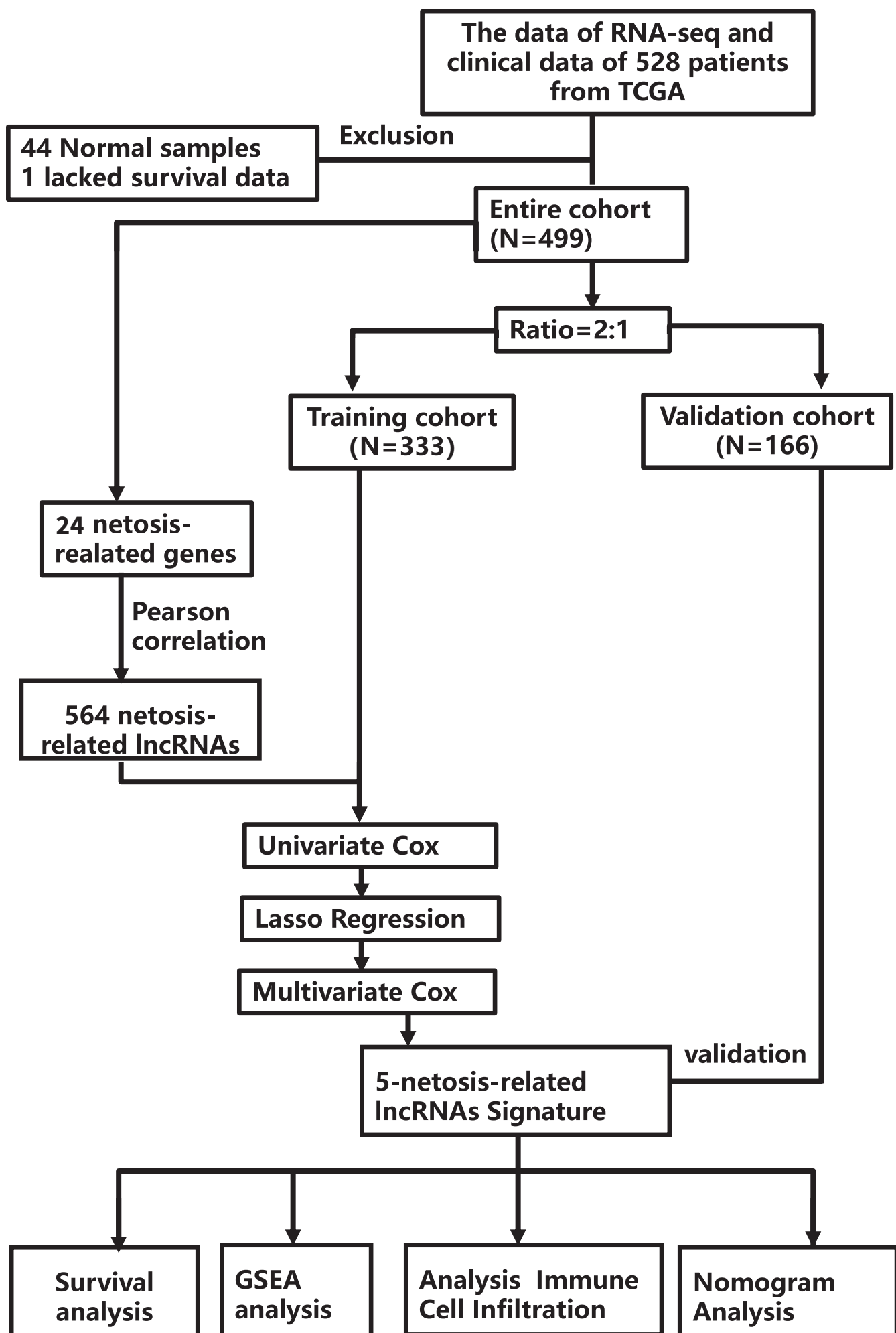

Supplement: Supplementary 2 — Figure S1: the flow chart of our study. Figure S2: the effect of LINC00426 on nasopharyngeal carcinoma cell lines. (A) Cell proliferation assays showed that LINC00426 overexpression did not affect the viability of CNE1 cells. (B) Plate colony formation assay showed that LINC00426 overexpression did not affect the ability of colony formation in CNE1 cells. (C) Cell proliferation assays showed that LINC00426 overexpression did not affect the viability of SUNE1 cells. (D) Plate colony formation assay showed that LINC00426 overexpression did not affect the ability of colony formation in SUNE1 cells. Wound healing assay showed that LINC00426 overexpression did not affect the ability of migration both in CNE1 (E) and SUNE1 (F) cells. [file 3191474.f2.zip › 3191474.f2/fig S1.pdf]

A

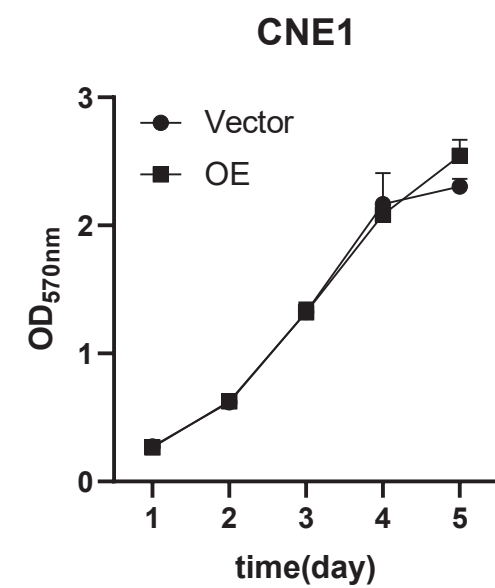

B

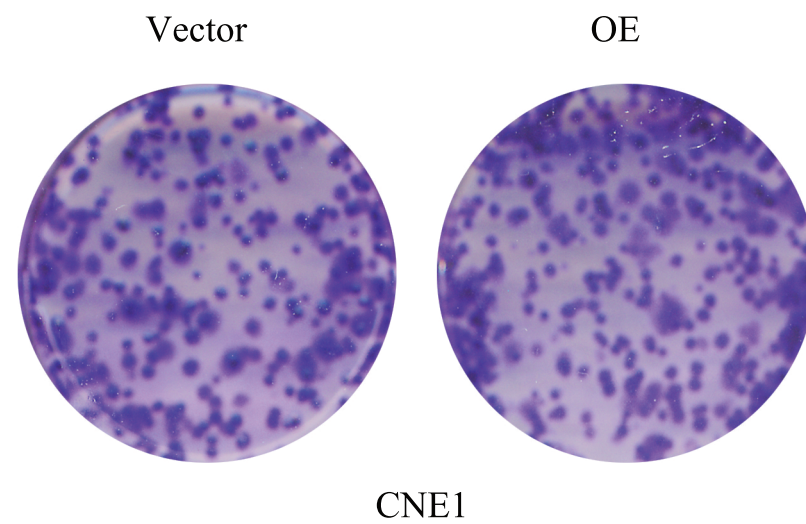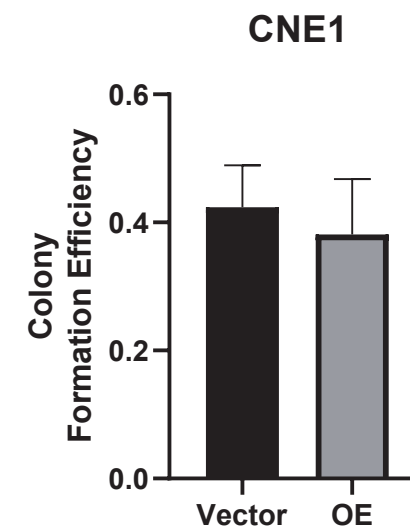

C

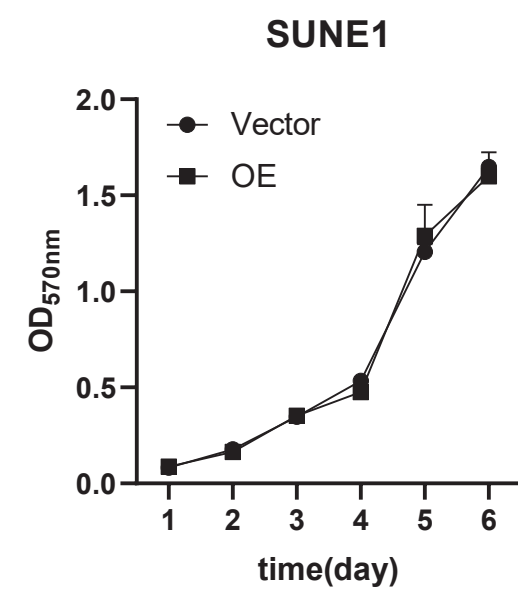

D

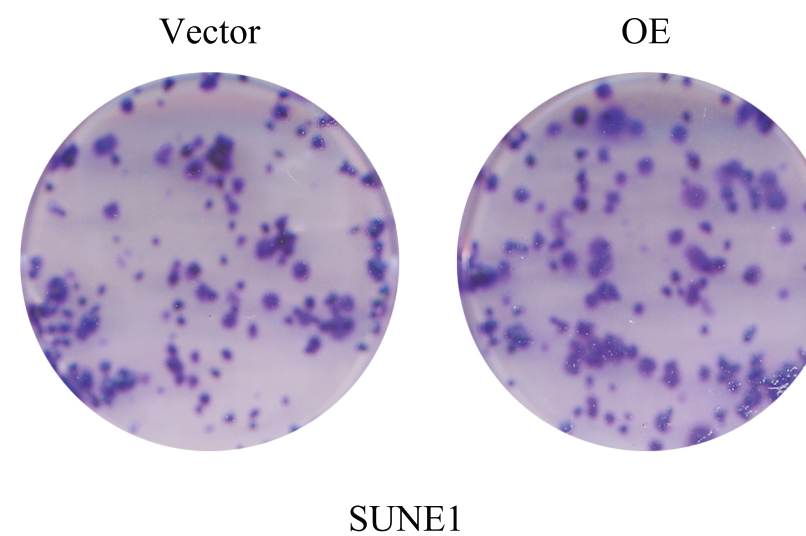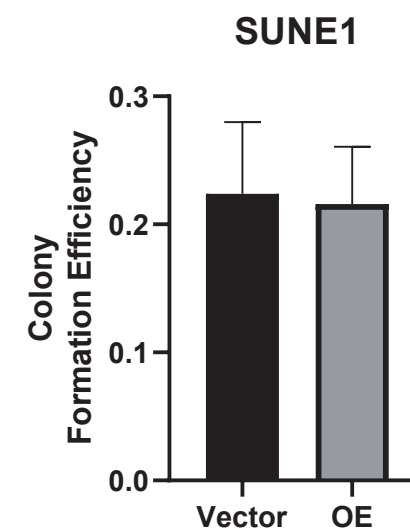

E

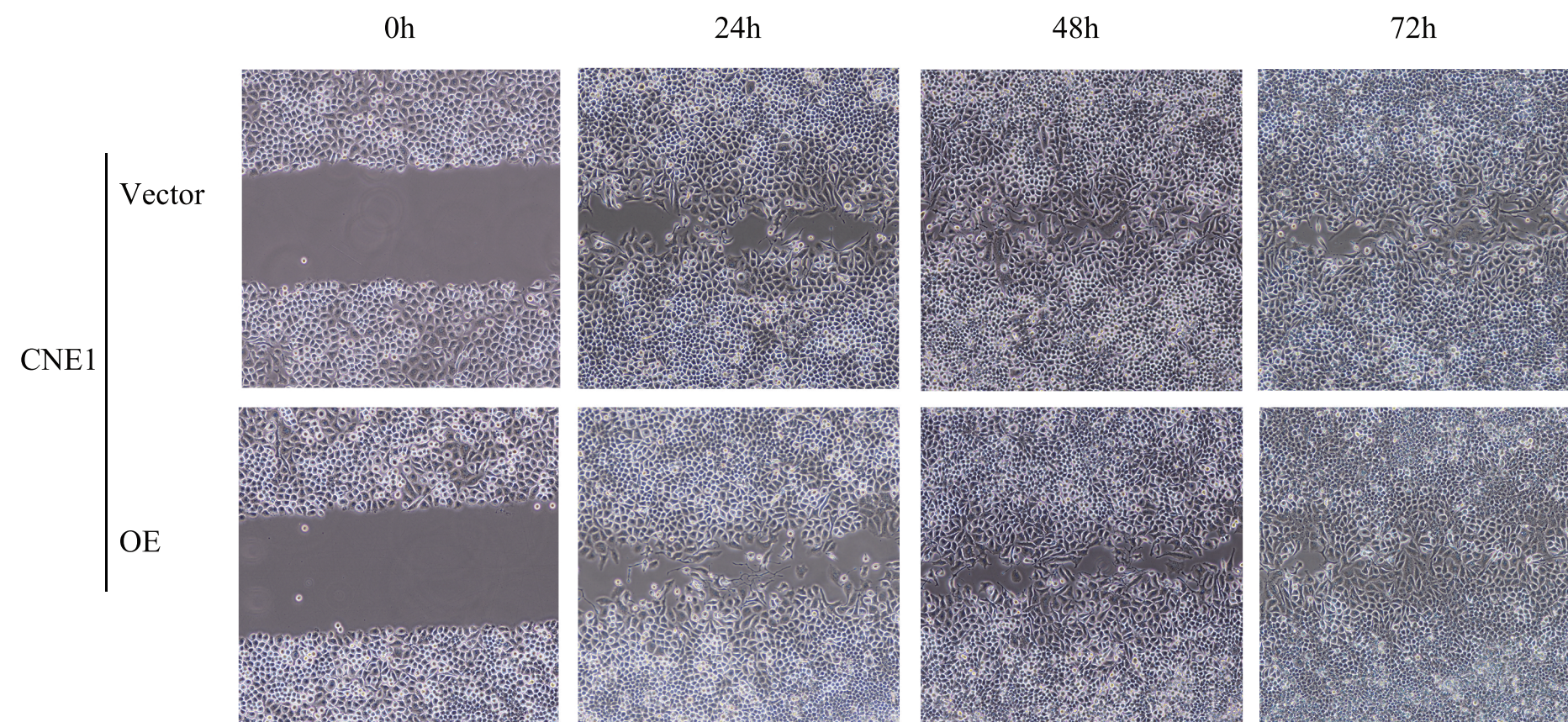

F

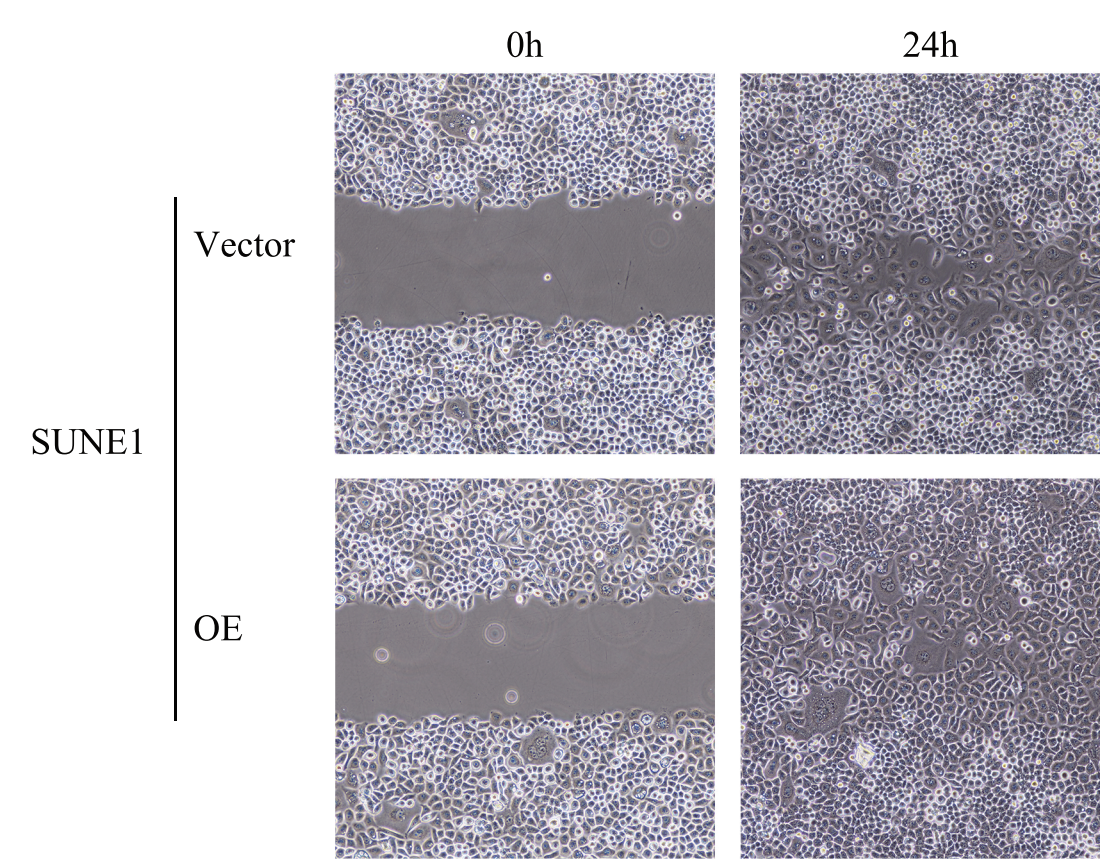

Supplement: Supplementary 2 — Figure S1: the flow chart of our study. Figure S2: the effect of LINC00426 on nasopharyngeal carcinoma cell lines. (A) Cell proliferation assays showed that LINC00426 overexpression did not affect the viability of CNE1 cells. (B) Plate colony formation assay showed that LINC00426 overexpression did not affect the ability of colony formation in CNE1 cells. (C) Cell proliferation assays showed that LINC00426 overexpression did not affect the viability of SUNE1 cells. (D) Plate colony formation assay showed that LINC00426 overexpression did not affect the ability of colony formation in SUNE1 cells. Wound healing assay showed that LINC00426 overexpression did not affect the ability of migration both in CNE1 (E) and SUNE1 (F) cells. [file 3191474.f2.zip › 3191474.f2/fig S2.pdf]
